# The Protein Corona as a Confounding Variable of Nanoparticle-Mediated Targeted Vaccine Delivery

**DOI:** 10.3389/fimmu.2018.01760

**Published:** 2018-08-02

**Authors:** Matthias Bros, Lutz Nuhn, Johanna Simon, Lorna Moll, Volker Mailänder, Katharina Landfester, Stephan Grabbe

**Affiliations:** ^1^Department of Dermatology, University Medical Center, Johannes Gutenberg University, Mainz, Germany; ^2^Max Planck Institute for Polymer Research, Mainz, Germany

**Keywords:** nanocarriers, cancer vaccines, immunotherapy, protein corona, cell-specific targeting

## Abstract

Nanocarriers (NC) are very promising tools for cancer immunotherapy. Whereas conventional vaccines are based on the administration of an antigen and an adjuvant in an independent fashion, nanovaccines can facilitate cell-specific co-delivery of antigen and adjuvant. Furthermore, nanovaccines can be decorated on their surface with molecules that facilitate target-specific antigen delivery to certain antigen-presenting cell types or tumor cells. However, the target cell-specific uptake of nanovaccines is highly dependent on the modifications of the nanocarrier itself. One of these is the formation of a protein corona around NC after *in vivo* administration, which may potently affect cell-specific targeting and uptake of the NC. Understanding the formation and composition of the protein corona is, therefore, of major importance for the use of nanocarriers in vaccine approaches. This Mini Review will give a short overview of potential non-specific interactions of NC with body fluids or cell surfaces that need to be considered for the design of NC vaccines for immunotherapy of cancer.

## Introduction

Immunotherapy of tumors has hit every day clinical practice in formerly hard-to-treat cancers due to the introduction of immune checkpoint modulators that block inhibitory, surface expressed molecules by antibodies (1). However, the use of antibodies against checkpoint inhibitors is not specific for a tumor antigen, since it reactivates pre-existing tumor immunity rather than priming novel T cell responses. This may result in insufficient clinical responses and in immune-related side effects due to unwanted autoimmunity in a substantial number of patients (2). The induction of tumor antigen-specific immunity remains a major goal of cancer therapy, targeting either overexpressed proteins or neoantigens that are unique to the individual tumor (3).

Tumor antigen-specific immunotherapy requires the delivery of the antigen—either as peptide, protein, DNA, or mRNA—to the correct cell type (4). Thus, targeting of antigen-presenting cells (APC), and concomitant induction of an appropriate APC activation status that enables immunogenic antigen presentation, is crucial for the success of therapeutic vaccination approaches (5). Nanotechnology holds great promise to transfer a packaged, protected cargo (antigen plus adjuvant) in high concentrations into the desired cell type by using appropriate nanocarriers (NC) (6). Indeed, vaccination studies using NC have demonstrated their great potential as universal vaccine platforms (7). Numerous strategies for specific targeting of NC to APC have been pursued, including the use of antibodies or their derivatives, natural ligands for receptors on the APC surface, aptamers, cystine knot proteins, or by modifying biophysical characteristics of the NC such as size and surface charge.

**Figure 1:**
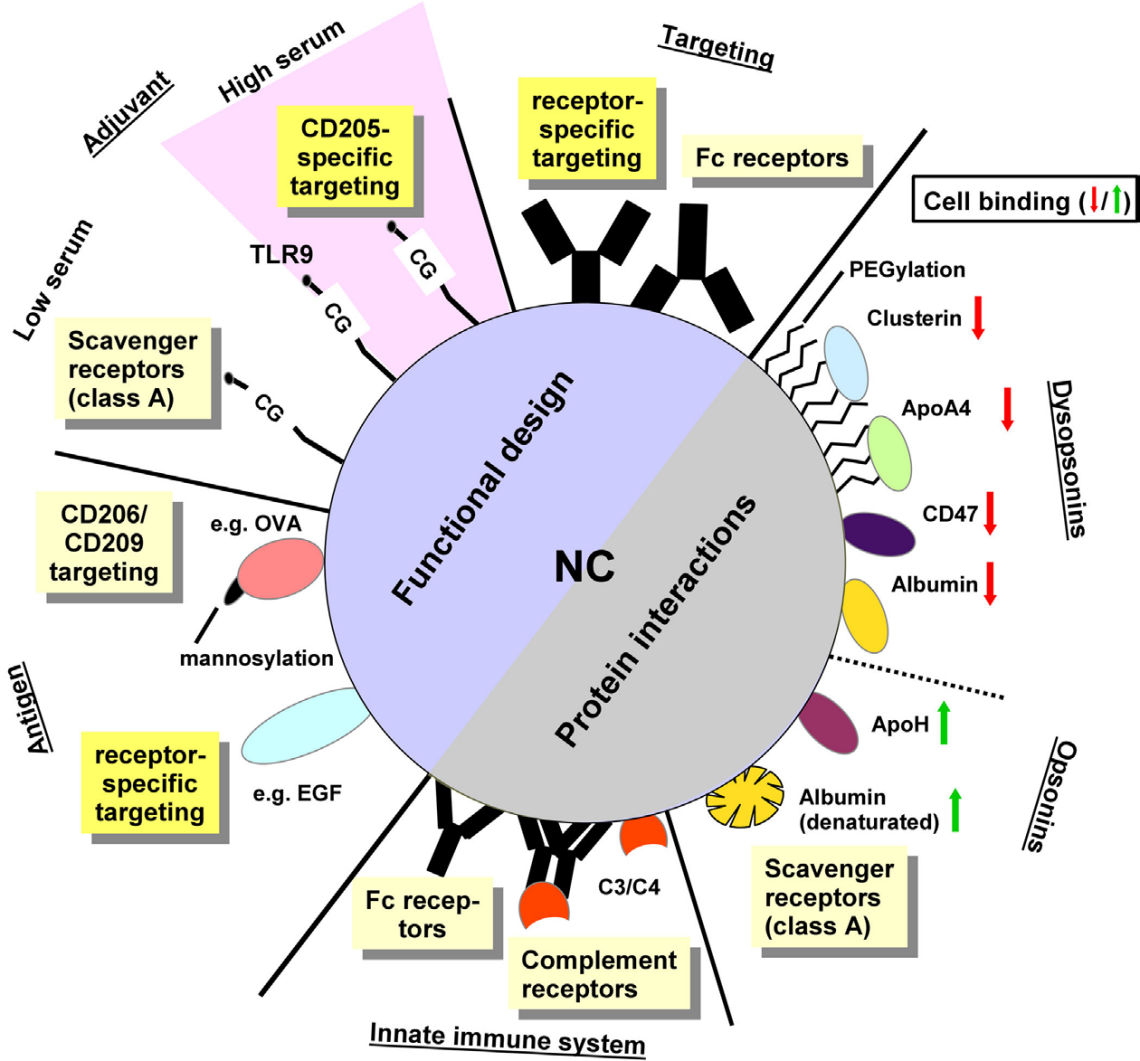
The functional design of NC and their protein interactions determine the character of cellular binding. Functional design: antibodies specific for receptors expressed by the target cell type are supposed to mediate cell type-specific targeting. In case of non-directed conjugation, the exposed Fc portion may result in binding to Fc receptor expressing cells. The adjuvant itself may mediate receptor specific binding as reported for toll-like receptor 9-activating CpG-rich oligonucleotides which target CD205 *in vivo*. Under standard culture conditions *in vitro*, however, oligonucleotides engage class A scavenger receptors (SR). The antigen may exert receptor-specific targeting, e.g., when mannosylated or in case of using a protein which constitutes a genuine receptor ligand. Protein interactions: recognition of the NC surface by components of the innate immune system like natural antibodies may yield Fc receptor binding and classical complement pathway activation. Direct recognition of the NC surface may trigger lectin-dependent/alternative complement pathways. Surface-deposited active C3 and C4 fragments mediate binding of complement receptors. Moreover, parameters of the NC surface like charge and hydrophobic/hydrophilic state determine the composition of the protein corona as well which in turn regulate subsequent cellular binding: albumin when adsorbed onto the NC surface in a denatured state enhances NC binding to SR. NC-adsorbed ApoH also elevates cellular binding. In contrast to these “opsonins” other factors like ApoA4, and (native) albumin as well as the “don’t eat me” signal protein CD47 and proteins found accumulated in the protein corona of PEGylated NC (e.g., clusterin) serve to reduce cellular interactions, and therefore were termed dysopsonins. The role of nanocarrier functionalizations and protein corona constituents for cellular binding is explained in more detail in Table S1 in Supplementary Material.

However, appropriate targeting of systemically applied NC to APC can be affected by unintended interactions of the NC surface with components of blood plasma (8) and/or with cell surface structures (9) that are unrelated to the specific targeting structure. The “protein corona” around NC may affect their organ-specific or cell type-specific trafficking as well as endocytosis and/or functional properties of the NC (10). Most importantly, the protein corona has been shown to interfere with targeting moieties used to induce receptor-mediated uptake of the NC, both inhibiting (11) and enhancing (12) internalization by specific cell types. Moreover, the protein corona is taken up by the target cell, which may alter their function. In this review, we will address various properties of the NC cargo and of the protein corona for targeted delivery of nanovaccines (Figure 1; Table S1 in Supplementary Material).

## Nanovaccines

Conventional vaccines that include a tumor antigen and an adjuvant do not specifically address specific types of APC. In addition, both components may dissociate and cause unwanted side effects. On the one hand, uptake of an antigen in the absence of an adjuvant by endocytic/phagocytic APC, but also by tumor-promoted myeloid-derived suppressor cells and tumor-associated macrophages (13) may cause tumor immune tolerance. On the other hand, stimulation of APC by an adjuvant alone may promote autoimmune reactions (14).

In general, nanovaccines can facilitate co-delivery of antigen and adjuvant. Earlier studies have shown that the stimulatory activity of a given adjuvant was enhanced when applied as a particulate formulation (15). For example, CpG-rich oligonucleotides, which engage endo/lysosomal Toll-like receptor 9 (TLR9) and are employed in clinical trials to boost anti-tumor responses (16), on an equimolar base exerted much stronger APC stimulation when coupled to a NC (15). Moreover, more recently small molecular TLR7/8 imidazoquinoline agonists were shown to be effective vaccine adjuvants when coupled to nanogels that drain lymph nodes, whereas they failed to trigger an immune response against co-injected antigen when applied in soluble form (17, 18).

For a number of nanovaccines containing antigen plus adjuvant, endocytic uptake by myeloid immune cell types has been reported, termed “passive targeting” (19). However, to prevent competitive uptake by unwanted phagocytically active myeloid cells (20), a specific targeting of APC which is capable to induce a (primary) anti-tumor response is essential. In this regard, dendritic cells (DC) are in the focus of nanovaccine development (21) since they are potently capable of priming naïve T cells (22). Most DC subpopulations present exogenous antigens rather exclusively *via* MHC-II. In order to obtain a profound antigen-specific CD8^+^ T cell response to directly kill malignant (or infected) cells, current approaches aim to target cross-presenting DC subpopulations (23, 24) which in mouse express surface receptors like CD205 (25), CLEC9a, and XCR1 at high levels (26). To this end, natural ligands of these surface receptors including mannose (27) and XCL1 (28) were successfully tested for DC targeting. As an alternative, receptor-targeting antibodies have been used (25, 29).

The surface marker which is used to target specific cell populations can also trigger uptake and may determine the intracellular route and ultimately the effectiveness of immune activation (30, 31). For example, using CD205 as a targeted surface marker seems to be favorable (32) as it enhances cross-presentation on MHC-I but also has a high amount of antigen peptides presented on MHC-II (25). We could recently show that a nanocarrier which co-delivered the model antigen ovalbumin (OVA) as well as an adjuvant (CpG-rich oligo) and was further decorated with a CD205-targeting antibody, yielded profound therapeutic activity in a mouse B16-OVA tumor model (12). In contrast, treatment of tumor-burdened mice with a nanovaccine formulation that lacked the DC-targeting antibody had no therapeutic effect. Interestingly, CD205-targeted delivery does not always accumulate antigens to DC when compared to mannose targeting (33). Thus, intracellular processing is as important as the vaccine dose that is taken up. The speed of internalization has also been suggested to play a role as in some studies slower internalization may favor better the preservation of MHC-I epitopes (33). It has been hypothesized that early endosomes that are involved in slow uptake processes have a lower concentration of proteases and thus avoidance of late endosomes seems to be favorable in this context. Certainly, lysosomal degradation occurs later with slower transport processes and the amount of peptides not totally degraded should be higher if the transport toward the lysosome is reduced. Other interesting and promising surface targets are CD40, Clec9a, and Clec12a since they have been shown to change intracellular trafficking (24). What we lack at this stage is a well-coordinated comparative study demonstrating the effectiveness of these different targeting vaccines in one animal model as most studies only imply none versus targeted antigens or compare two targeting pathways.

Altogether, these findings support the rationale to design multifunctional nanovaccines. However, we and others also observed that the largest fraction of systemically applied nanovaccine accumulated in the liver, irrespective of its formulation (34), which suggests general involvement of yet unknown factors that interfere with cell type-specific targeting.

## The Protein Corona Around NC as a Confounding Variable for Effective Vaccine Delivery

Despite their many advantages, NC are complex molecules that may interact with serum proteins and other components of body fluids in an unexpected manner, which may significantly alter their efficacy as vaccine carriers. One of these non-intended interactions is the spontaneous (ir-)reversible deposition of proteins onto the NC surface in complex fluids, which is modified by multiple parameters, either related to the NC source or the composition of the protein environment. Some basic principles of this process were elucidated by mimicking *in vivo* NC protein interactions *via in vitro* incubation with biological fluids (35). However, additional physiologically relevant factors (e.g., stability and dynamics of protein coronae under shear stress during passage through the blood) are still poorly understood (36). Nevertheless, some relevant determinants of protein corona formation around NC have been defined and verified in murine models *in vivo*.

### Physico-Chemical Properties and Surface Functionalization of NC as Determinants of Protein Corona Formation

#### Hydrophilic Versus Hydrophobic Surfaces

Due to their large surface areas in relation to their volume, nano-sized materials are highly affected by surface interactions with body fluids such as plasma or lymph (37). The chemical composition of the NC at the surface enables protein binding through hydrogen bonds, hydrophobic interactions, electrostatic interactions, and π-π stacking (38). Therefore, NC surface chemistry needs to be optimized to prevent particle aggregation under biologically relevant conditions. For instance, Lundqvist et al. compared plain polystyrene NC with surface carboxy- or amine-modified ones and identified both proteins that were common on all types of NC as well as some that were specifically enriched on each of the surfaces (39). In general, hydrophobic particles including non-functionalized polystyrene NC are not well-dispersible and stable in water or even protein-rich solutions over time, as they require surface active agents (surfactants) to reduce the large surface tension between the two phases (40). Usually, these surfactants are small molecular detergents or amphiphilic (co-)polymers that stabilize the interface molecularly. However, proteins which usually provide a hydrophilic surface and a hydrophobic core can also partially expose some of their hydrophobic residues which might compete with the surfactants and replace them irreversibly. Therefore, a well-defined and stable interface with immobilized surfactants on a hydrophobic carrier surface would be advantageous to prevent NC protein aggregation (41). On the other hand, hydrophobicity can also be utilized to control protein adsorption on the nano-bio-interface specifically, as demonstrated by Zhang et al. who covalently deposited (co-)polymers of varying amphiphilicity on gold NC and observed a variation in protein adsorption affording tailored cellular NC uptake (42).

#### Surface Charge

Besides hydrophobic interactions, proteins can undergo also charge-driven binding to the NC interface (as most protein surfaces are composed of charged amino acids) (43). Albumin as one of the most abundant proteins in blood plasma has a slightly negative net charge (44) and, therefore, instantaneously interacts with positively charged NC (8, 45). For instance, multi-angle dynamic light scattering in human blood plasma was applied as a highly sensitive method to monitor the binding of albumin on cationic nanohydrogel particles (46). Deposition of albumin onto nanogels was prevented by loading the nanogels with negatively charged siRNA oligonucleotides for RNA interference therapy and, thus, neutralizing the net charge of the nanogels and enhancing their circulation properties in the blood stream.

Yet, charge-neutral polymers can still adsorb proteins and influence the carriers’ *in vivo* performance. As an example, iron oxide NC coated with dextran yielded deposition of activated complement C3 and triggered complement receptors (CR)1/2-mediated B cell targeting which was further exploited for the treatment of allergic immune responses (12).

#### PEGylation of NC

To minimize protein interaction with polymer coatings and biomaterials, Whitesides and co-workers investigated different chemical structures on self-assembled monolayer interfaces and identified four basic principles [so-called “four Whiteside’s rules”] (47) that efficiently suppress protein adsorption (48, 49): (1) hydrophilicity, (2) no charges, (3) no hydrogen bond donors, and (4) only hydrogen bond acceptors. All these characteristics are fulfilled by poly(ethylene glycol) (PEG), one of the most frequently used polymer NC coatings to minimize—but not always completely abolish (see Composition of the Biological Fluids as Determinants of the NC Protein Corona)—protein adsorption but guaranteeing a stealth-like behavior for enhanced circulation properties after systemic administration (50–52). To that respect, we have recently shown that for PEGylated polystyrene NC the stealth effect is not due to the avoidance of protein adsorption, but rather the adsorption of specific proteins like clusterin or apolipoprotein A4 (ApoA4) (53). Still, the degree of PEGylation on the nano-biointerface as well as PEG density can modify the protein corona and its NC performance under biological conditions. For instance, Kataoka and co-workers recently showed that tethered PEG density with highly squeezed PEG chains on the interface of pDNA-polyplexes assured higher circulation properties to improve pDNA delivery (54, 55). For site-specific targeting of NC with ligands to manage selective interaction with the ligand-corresponding receptor, PEGylation is often the only way to reduce additional uncontrolled protein corona formation, which would counteract with the targeting groups (56). However, in some cases too dense PEGylation can also entrap a targeting ligand inside the PEG interface and suppress its interaction with its target receptor (57). Yet, in these cases PEG backfilling with shorter PEG chains can help to both reduce massive protein corona formation as well as assuring ligand accessibility to their receptors (58). While a better understanding of the PEGylation process on the NC surface has become increasingly evident, controversial concern of use of PEG for biomedical purposes (59) has motivated the development of alternative stealth-like polymers (60) which might result in a better controllable protein corona formation on NC after administration into a biologically relevant environment.

#### Endotoxin Contamination

The formation of a NC protein corona was found to be further modulated by prior adsorption of the Gram-negative bacteria cell wall component lipopolysaccharide (LPS) (61). LPS is a frequent contaminant of proteins used for nanovaccine generation (e.g., antigen and targeting antibody), and of non-sterile lab environments. LPS was reported to bind various types of NC both *via* charge-driven interactions (negatively charged phosphate head groups interact with cationic nanoparticles) and by hydrophobic interactions (LPS lipid regions interact with hydrophobic domains on the nanoparticle) (62). LPS-contaminated NC stimulated inflammatory responses by co-incubated toll-like receptor 4-expressing immune cells (61). These observations underscore the absolute requirement to test NC for endotoxin contaminations prior to functional testing.

### Composition of the Biological Fluids as Determinants of the NC Protein Corona

Besides the physicochemical properties of the NC, the composition of the biological fluid it is immersed into is another relevant factor in the formation of the protein corona (63). In terms of *in vitro* studies, fetal bovine serum, human plasma, or serum are mainly utilized in order to investigate protein-NC interactions and their functional effect on the cellular level (64). The difference between serum and plasma is highly significant in terms of corona composition and ultimately affects the interaction of coated NC with immune cells (65). This difference is caused by the preparation procedure as blood is either naturally coagulated (serum) or supplemented with an anti-coagulant (plasma). Here, it has to be noted that also the choice of the anti-coagulant being either citrate, heparin, or EDTA additionally influences corona formation (63) and cellular outcome (66).

Bringing NC from pre-clinical studies toward the clinical application bears additional challenges. Differences in the corona composition between mice and humans (67) as well as inter-individual variations in plasma protein composition, including dietary factors that affect, e.g., serum lipoprotein composition (“personalized protein corona”) have been recognized (68). On top of this, several reports could show that *in vitro* studies cannot fully reflect the situation *in vivo* (69, 70). The interaction of NC in blood flow is highly dynamic, may be altered by shear stress and hereby strongly alter the composition of the protein corona pattern (71). Based on this, a better understanding of the *in vivo* protein corona formation and composition is still under investigation and is needed to eventually tune the NC properties for targeted cellular interaction.

## Cellular Receptors for NC Corona Proteins

The plasma protein corona around NC can significantly alter their biological behavior *in vivo* (9) and also affect specific targeting moieties that are being used to target NC to specific organs or cell types (11). In many cases, the protein corona may impair the targeting structure on the NC from binding to its receptor on the target cell. This may indeed occur much more frequently than reported, since unsuccessful attempts for targeted delivery of NC are typically not published. In some cases, however, NC corona proteins may also enhance binding of the NC to target cells which bear receptors that recognize specific NC corona proteins (12). As outlined below, among the receptors that bind NC surface molecules in a non-specific manner are CRs, scavenger receptors (SR), immunoglobulin receptors, and lipoprotein receptors. Although not formally shown yet, other phagocytic receptors (72) may also be involved in NC recognition by leukocytes and endothelial cells.

### Fc Receptors

Fc receptors bind immunoglobulins *via* their constant (Fc) region (73). There are specific receptors for IgG (FcγRI [CD64]), (FcγRIIA [CD32]), (FcγRIIB [CD32]), (FcγRIIIA [CD16a]), (FcγRIIIB [CD16b]), IgA (FcαRI [CD89]), and IgE (FcεRI and FcεRII [CD23]). These receptors bind immunoglobulins with differential affinity and also modify the functional state of the receptor-bearing cell. Especially for Fcγ receptors, different biological functions of various receptors are known, ranging from antibody-dependent cell-mediated cytotoxicity (73), to phagocytosis, and cell activation (74), or inhibition of cell activity (75). Receptor-specific antibodies are commonly used to enable targeting of NC. They are typically coupled to the NC surface in a non-oriented form (73). Thus, it is often arbitrary whether the antigen-binding Fab or the Fc portion of the molecule is exposed to the outer surface of the NC, resulting in potential binding of the antibody-coated NC to FcR carrying cells *in vivo* (mostly macrophages and liver endothelial cells) (73). Likewise, immunoglobulins derived from plasma may also bind to NC, either in an epitope-specific form (thus “opsonizing” the NC) or *via* non-specific adsorption (76). It is tempting to speculate that this non-epitope-specific binding of antibody-coated NC may interfere with any intended specific targeting of the NC *via*, e.g., the antigen-binding epitope of an NC-coupled antibody. In contrast, immunoglobulin binding to PLGA nanocarriers has also been demonstrated to inhibit non-specific interaction with endothelial cells in human blood flow (77). For clinical applications, it will be imperative to overcome uncontrolled FcR-mediated effects of antibody-targeted NC by using either antibodies coupled *via* the Fc part to the NC to prevent its unintended binding to the FcR (78) or antibody derivatives that lack the Fc portion (79).

### Complement Receptors

Complement receptors (CR) are expressed mainly by leukocytes and bind bacteria and other structures opsonized by complement factors as a consequence of classical, alternative, or lectin-mediated complement pathway activation (73). Opsonized material is recognized by CR 1–4. CR1 (CD35), CR3 (CD11b/CD18), and 4 (CD11c/CD18) which mediate phagocytosis by mononuclear cells, whereas CR2 (CD21) is present only on B cells and serves as a co-receptor (80, 81). All types of NC investigated by us that carry glyco-structures on their surface (e.g., dextran and starch) avidly bound and activated the lectin-dependent complement pathway, whereas inorganic NC generally failed to do so (12). Ligation of C3-coated NC by CR2 resulted in efficient binding of iron oxide-dextran NC by murine B cells, resulting in specific targeting of these NC to B cells *in vivo* (12). C3/CR2-mediated B cell engagement of the NC significantly surpassed antibody-mediated targeting, as NC that were additionally coated with an anti-CD205 antibody that is recognized by DC still bound much more abundantly to B cells than to DC *in vivo*. Thus, plasma protein corona components may re-direct NC to certain cell types *in vivo*. This effect can be exploited in an immunotherapeutic fashion, as dextran-iron oxide NC that contained an antigen plus CpG as an adjuvant could be used to efficiently treat B cell-mediated hypersensitivity reactions such as allergic asthma and anaphylaxis.

### Scavenger Receptors

Scavenger receptors serve to endocytose diverse polyanionic ligands including modified endogenous (lipo)proteins like oxidized low-density lipoprotein, but also pathogen-derived molecular patterns and endogenous misfolded proteins (82). Low-density lipoproteins are regularly identified in the protein corona of different nanoparticles (53, 83). Due to their interaction with different toll-like receptors, and their association with intracellular signaling complexes like mitogen-activated protein kinases, SR engagement was shown to alter the cellular activation state of DC (84) and macrophages (85). Class A SR (SR-A) that contain a collagen domain were shown to bind negatively charged surfaces on dextran NC (86), polystyrene NC (87), silica NC (88), and superparamagnetic iron oxide NC (89) under standard culture conditions *in vitro*, i.e., at low serum concentration and in the absence of complement and immunoglobulin (53, 83).

Due to the compensatory capacity of single SR-A, binding of NC to this class of receptors is validated most often in blocking studies using fucoidan, Poly(I), and dextran sulfate as competitive high affinity SR-A ligands (90). In this regard, dextran sulfate-based NC were shown to retain their SR-A binding affinity also *in vivo*, and were used to target activated macrophages in a model of murine arthritis (91).

Negatively charged NC surfaces, such as NC conjugated to short linear (anionic) oligonucleotides were efficiently internalized *via* SR-A *in vitro* (92). Consequently, SR-A-mediated internalization of oligonucleotide-conjugated NC was exploited for efficient transfer of drugs and siRNA into different cell types (93). Pre-incubation of such NC with serum dose-dependently inhibited cell binding, presumably due to shielding of the negatively charged oligonucleotides by yet unknown serum factors (94).

## Other Corona Proteins That Affect NC Adsorption to Cells

Besides corona proteins that mediate binding to classical phagocytosis receptors, other corona proteins also affect the cellular uptake of NC.

### Dysopsonins

The main characteristic of “stealth” NC is their reduced interaction with phagocytic cells, which results in a prolonged blood circulation time (95). Overall, stealth NC show less protein adsorption, however, protein corona formation cannot be completely prevented (96). Thus, in general, non-recognition of NC by immune cells is not only due to low amounts of proteins adhering to surfaces but can also dependent on the abundancy of certain corona proteins. Actually, we have identified distinct proteins which inhibit cellular interactions and hereby mediate stealth behavior (63, 97). Those proteins are refereed as “dysopsonins”, of which albumin and clusterin (apolipoprotein J) are the most prominent examples. Clusterin has been demonstrated to be required for the stealth effect of poly(ethylene glycol)- and poly(phosphoester)-coated polystyrene NC (53). Albumin, the most abundant protein in serum (98), is a prominent constituent of the protein corona of many types of NC (99). Takeuchi and co-workers (100) demonstrated recently that albumin specifically adsorbs to polymeric nanogels after *in vivo* administration, creating an albumin-rich corona which prolonged blood circulation.

Thus, pre-coating of different types of NC with albumin can improve their circulation half-life and biocompatibility (101). However, when misfolded, albumin coating of NC may also shorten their plasma half-life. Indeed, albumin underwent conformational changes of its alpha-helical domains after adsorption to layered silicate NC (102) and polystyrene NC with a cationic, amino-modified surface (103). In both studies, NC adsorbed with misfolded albumin effectively bound SR-A *in vitro*. Likewise, albumin adsorption to inorganic NC (104–106) also resulted in an unfolding of alpha-helical domains, and similar conformational changes were also reported for other serum proteins like fibrinogen, gamma-globulin, histone, and insulin when adsorbed onto gold NC (107). Further studies need to elucidate whether NC may unintendedly bind SR *in vivo* due to conformationally altered serum factors within their protein corona.

### Apolipoproteins

In general, apolipoproteins were identified in high amounts on the surface of various NC formulations (108, 109). For example, ApoE was enriched on the surface of NC coated with the nonionic surfactant polysorbate 80 and hereby enabled the transport of NC across the blood–brain barrier *via* receptor-mediated endocytosis (110). Additionally, recently adopted immuno-mapping techniques (111) offer the possibility to determine functional cell receptor-binding epitopes of corona proteins. Here, it was found that SiO_2_ NC are covered by ApoB100 which allows a recognition of NC *via* low-density lipoprotein receptor (112). Moreover, in another study Ritz and coworkers identified a variety of different proteins within the corona of differentially surface-functionalized polystyrene NC, and could correlate their relative abundance with an enhanced or decreased uptake by human mesenchymal stem cells (35). As demonstrated in that study, ApoA4 and C3 were shown to decrease unspecific cell interaction whereas ApoH enhanced cellular uptake.

### “Don’t Eat Me” Signals

Viable cells, most notably erythrocytes and platelets, express surface receptors like CD31, CD47, and CD200 that interact with counter-receptors on myeloid immune cells to prevent their cytolysis [reviewed in Ref. (113)]. Furthermore, living cells show extensive sialic acid modifications of glycoproteins. Presentation of such “don’t eat me” signals has been used to prevent phagocytic clearance of NC. CD47 is ubiquitously expressed and binds SIRPα that is predominantly expressed on phagocytically active leukocytes (114). SIRPα engagement results in the activation of phosphatases that inhibit phagocytic activity. Rodriguez et al. (115) demonstrated that CD47-derived peptides coupled to polystyrene beads reduced their uptake by macrophages, and prolonged their circulation in mice. In line, different types of NC (polystyrene, PLGA) conjugated with an ICAM-1 targeting antibody for endocytic uptake by activated endothelial cells showed clearly reduced unspecific liver accumulation when conjugated in addition with CD47 (116). In a different approach, NC were coated with cell membranes derived from red blood cells to exploit their endogenous high level surface expression of CD47 and other “don’t eat me signals” [reviewed in Ref. (117)]. This concept has been broadened by transferring membranes of specific leukocyte populations to make use of the cell type-specific properties of their surface receptors like mediating cell–cell adhesion and homing behavior.

## NC Design—Avoid or Exploit the Protein Corona?

Concerning the design of APC targeting nanovaccines, it is necessary to take into account potential intrinsic receptor binding properties of antigen and adjuvant. For example, short oligonucleotides which engage DNA binding danger receptors like TLR9 or STING and thereby activate APC (118) were demonstrated to effectively engage SR-A in a serum-poor environment (94). *In vivo*, however, CpG-rich oligos engage CD205 which is highly expressed by CD8^+^ DC in mouse (119). We showed that nanovaccines conjugated to this adjuvant retained both their CD8^+^ DC binding and activating properties *in vivo* (120). Proteins used as a source of antigen may be recognized by receptors if they constitute genuine ligands (e.g., epidermal growth factor) or may bind *via* a protein modification as demonstrated for OVA which is endocytosed *via* the mannose receptor due to mannosylation of the protein (121). In order to prevent interactions of antigen/adjuvant with cellular receptors (or serum components), nanocapsules may be preferable to shield and to protect the cargo of a nanovaccine (122). If the intrinsic binding properties of cargo components support the intended NC targeting other types of NC that expose their cargo may be employed.

To achieve cell type-specific targeting either antibodies or their fragments, synthetic ligands (e.g., aptamers, DARPins, and cystine-knot miniproteins), or natural ligands of endocytic surface receptors highly expressed by the target APC may be used. However, depending on the orientation of a NC-coupled targeting antibody binding of the exposed Fc part to Fc receptors is possible (73) and may limit cell type specificity. Similarly, a conjugated receptor ligand may bind different receptors as exemplified for mannose-derived oligosaccharides which may engage both the mannose receptor and DC-SIGN (123). Consequently, the efficacy and specificity of NC binding, uptake and subsequent biological effects need to be tested using cell populations comprising also non-target cell types (e.g., human PBMC, mouse spleen, and liver cells).

To predict the *in vivo* behavior of a NC by *in vitro* assays in a more reliable manner, it is necessary to allow formation of a protein corona in a controlled way. One strategy is to minimize adsorption of serum factors to the NC surface which may affect its intended targeting properties (see Physico-Chemical Properties and Surface Functionalization of NC as Determinants of Protein Corona Formation). On the contrary, however, the composition of the protein corona itself may support the biological function of a nanovaccine. For example, we have recently demonstrated that a lectin surface coating of NC resulted in activation of the lectin complement pathway and enabled specific NC targeting to B cells *via* CRs (12). Thus, the protein corona may inhibit or enable cell type-specific targeting.

## Summary

In summary, NC are versatile tools to deliver a high amount of antigen plus adjuvant(s) in a targeted manner to APC. Here, we point toward a variety of interesting receptors like CD205 or Clec9A that can focus delivery toward favorable immunological readouts. However, NC are almost inevitably coated with a protein corona after exposure to blood plasma or lymphatic fluid. This plasma protein corona can affect the trafficking of the NC within the body as well as their cellular targeting and uptake to a significant extent, potentially resulting in loss of the desired effects as well as altered functional properties of the NC. Often, antibodies are used as targeting moieties; yet, the interaction of their Fc part with receptors of other cells represents an undesired mistargeting and should be avoided for nanovaccines. On the other hand, the protein corona may also be exploited to extend NC plasma half-life, e.g., by attracting or preadsorbing clusterin, thereby optimizing cell-specific targeting and immunotherapeutic effects, or even to direct NC to specific cell types or organs *in vivo* by exploiting (pre)adsorbed targeting moieties.

## Author Contributions

All authors contributed writing the manuscript of this Mini Review.

## Conflict of Interest Statement

The authors declare that the research was conducted in the absence of any commercial or financial relationships that could be construed as a potential conflict of interest.
