# Supplementary material for: The Protein Corona as a Confounding Variable of Nanoparticle-Mediated Targeted Vaccine Delivery
Source: Front Immunol. 2018 Aug 2;9:1760. doi: 10.3389/fimmu.2018.01760 (PMC6082927; doi:10.3389/fimmu.2018.01760)
Supplement: Supplementary file 1 [file Table_1.DOC]

Table S1. NC functionalization and non-specific protein interactions influence cellular binding.

| **Functional design** | **NC functionalization** | **NC behavior** | | **Reference** |
| --- | --- | --- | --- | --- |
|  |  | **Intended** | **Side effect** |  |
|  | non-directed conjugation of cell surface receptor targeting antibodies | cell-specific targeting |  | Kawasaki et al., 2014; Shen et al., 2016; Zeng et al., 2018 |
|  |  |  | binding of exposed Fc to Fc receptor bearing cells / Fc receptor mediated phagocytosis | Daeron, 2014 |
|  | immunostimulatory CpG-rich oligo-nucleotides | engagement of endo/lysosomal TLR9 to yield APC activation |  | Shirota and Klinman, 2014; Amoozgar and Goldberg, 2015 |
|  |  |  | at low serum (*in vitro*): binding of scavenger receptors class A / receptor-mediated endocytosis  (highly expressed by myeloid cell types) | Rosi et al., 2006; Li et al., 2017 |
|  |  |  | *in vivo*: specific targeting CD205 receptor / receptor-mediated endocytosis  (predominantly expressed by CD8a^+^ DC [mouse], and fractions of B cells) | Lahoud et al., 2012; Shen et al., 2016 |
|  | mannosylated proteins (e.g. oval-bumin) | source of antigen; required to elicit antigen-specific adaptive immune response |  | Macri et al., 2016; Moyer et al., 2016; Shen et al., 2016 |
|  |  |  | binding to CD206 (mannose receptor)  (mainly expressed by macrophages and conven-tional DC populations) | Burgdorf et al., 2006 |
|  |  |  | binding to CD209 (DC-SIGN)  (mainly expressed by DC and some macrophages) | van Liempt et al., 2006 |

| **Protein interaction** | **NC surface-dependent protein binding** | **NC behavior** |  | **Reference** |
| --- | --- | --- | --- | --- |
|  | ApoH (polystyrene NC) | enhanced unspecific cell binding | | Ritz et al., 2015 |
|  | albumin (misfolded after binding to different types of anorganic NC) | enhanced unspecific cell binding due to recognition by scavenger class A receptors | | Chakraborti et al., 2011, 2012; Podila et al., 2012; Mortimer et al., 2014; Fleischer and Payne, 2014 |
|  | natural antibodies recognizing NP surface moieties | binding of exposed Fc to Fc receptor bearing cells / Fc receptor mediated phagocytosis  in case of Fc cross-linking: activation of classical complement pathway / complement receptor mediated internalization  (complement receptors predominantly expressed by myeloid cell types [CR3, CR4] and B cells [CR1/2] | | Daeron, 2014; Caracciolo et al., 2015 |
|  | NP surface (i.e. lectin) triggers alternative/lectin dependent complement activation | complement receptor-mediated internalization | | Erdei et al., 2017; Shen et al., 2018 |
|  | NP surface PEGylation results in lower overall protein and may enhance relative abundance of  Clusterin  ApoA4 | reduced (unspecific) cell binding | | Bazile et al., 1995; Harris and Chess, 2003 |
|  |  |  |  | Schöttler et al., 2016a |
|  | Albumin (negatively charged; binds cationic NC) |  |  | Aggarwal et al., 2009; Mariam et al., 2017; Takeuchi et al., 2017 |
|  | CD47 (conjugated) | reduced binding/uptake by meyloid cells due to interaction with SIRPα receptor expressed by myeloid cells (`don´t eat me´ signal) | | Rodriguez et al., 2013; Kim et al., 2017 |
